# Intimate communications within the tumor microenvironment: stromal factors function as an orchestra

**DOI:** 10.1186/s12929-022-00894-z

**Published:** 2023-01-04

**Authors:** Bing Cheng, Qiang Yu, Wenyu Wang

**Affiliations:** 1 Guangdong Provincial Key Laboratory of Colorectal and Pelvic Floor Disease, The Sixth Affiliated Hospital of Sun Yat-Sen University, Guangzhou, China; 2 Guangdong Research Institute of Gastroenterology, The Sixth Affiliated Hospital of Sun Yat-Sen University, Guangzhou, China; 3 Cancer Precision Medicine, Genome Institute of Singapore, Agency for Science, Technology, and Research, Biopolis, Singapore; 4 Department of Physiology, Yong Loo Lin School of Medicine, National University of Singapore, Singapore, Singapore; 5 Cancer and Stem Cell Biology, DUKE-NUS Graduate Medical School of Singapore, Singapore, Singapore

**Keywords:** Tumor microenvironment, Cancer-associated fibroblast, Epigenetic regulation, Immunotherapy

## Abstract

Extensive studies of the tumor microenvironment (TME) in the last decade have reformed the view of cancer as a tumor cell-centric disease. The tumor microenvironment, especially termed the "seed and soil" theory, has emerged as the key determinant in cancer development and therapeutic resistance. The TME mainly consists of tumor cells, stromal cells such as fibroblasts, immune cells, and other noncellular components. Within the TME, intimate communications among these components largely determine the fate of the tumor. The pivotal roles of the stroma, especially cancer-associated fibroblasts (CAFs), the most common component within the TME, have been revealed in tumorigenesis, tumor progression, therapeutic response, and tumor immunity. A better understanding of the function of the TME sheds light on tumor therapy. In this review, we summarize the emerging understanding of stromal factors, especially CAFs, in cancer progression, drug resistance, and tumor immunity with an emphasis on their functions in epigenetic regulation. Moreover, the importance of epigenetic regulation in reshaping the TME and the basic biological principles underpinning the synergy between epigenetic therapy and immunotherapy will be further discussed.

## Background

Cancer is one of the main life-threatening diseases worldwide. Although substantial improvements have been achieved in early detection and treatment paradigms, tumor recurrence, metastasis, and therapeutic resistance remain the major challenges in almost all kinds of cancers. A better understanding of the underlying mechanism of tumor development and progression may provide opportunities for cancer treatment.

Genomic instability and mutation in cancer cells have been considered the fundamental driving characteristics during cancer progression; therefore, substantial studies have largely been restricted to the epithelial component of tumors. However, a tumor is not an island originating from cancer cells but rather a complex cellular ecosystem. The “seed and soil” theory was first proposed by Stephen Paget in 1889 to interpret the preferences of cancer cells when metastasizing to organs [1, 2]. For the first time, this theory emphasizes the importance of the host environment to the appearance of tumor metastasis in addition to the intrinsic properties of cancer cells. It also has important reference significance for understanding tumorigenesis, tumor progression, and drug resistance in cancer. The maintenance and progression of tumors are highly supported by the tumor microenvironment (TME), also termed the tumor stroma, which mainly includes fibroblasts, immune cells, the basement membrane, the extracellular matrix, and the vasculature [3]. As the most abundant cell type in the TME, cancer-associated fibroblasts, which transdifferentiate from their main precursors, normal fibroblasts, play pivotal roles in tumor progression. The interplay between cancer cells and CAFs, which affects tumorigenesis and tumor development from almost every aspect, has become increasingly unveiled in recent years.

Epigenetic regulations, including DNA methylation, histone modification, chromatin remodeling, and noncoding RNA regulation, change gene expression without affecting germline DNA sequences. In addition to genetic mutations, epigenetic dysfunction is recognized as an important hallmark of cancer. It has been widely recognized that epigenetic alterations can drive oncogenesis and promote cancer progression by extensively regulating the aberrant expression of cancer-associated and immune-related genes. In cancer cells, the transcription of proto-oncogenes is commonly boosted as a result of promoter hyperacetylation, while tumor suppressor genes are repressed by promoter hypoacetylation and DNA hypermethylation. Many cancers show a global loss of DNA methylation across the genome, with gains of DNA methylation primarily at CpG islands, suppressing genes that control cancer progression [4]. As one of the most important mechanisms, epigenetic dysfunction fundamentally reshapes the tumor microenvironment by altering the phenotypes of cancer cells, tumor-associated stromal cells, and immune cells. The reversibility of epigenetic modifications has enabled pharmacological interventions with potential therapeutic strategies as either monotherapy or in combination with other therapies. In particular, epigenetic agents have shown great potential for synergizing with cancer immunotherapy, such as immune checkpoint blockade (ICB), due to their potent ability to convert the TME into a more immunopermissive type.

In this review, we summarize the latest understanding of stromal factors, especially CAFs, in cancer progression, therapeutic resistance, and tumor immunity with a particular emphasis on their functions in epigenetic regulation. In addition, the importance of epigenetic regulation in reshaping the TME and the basic biological principles underpinning the synergy between epigenetic therapy and immunotherapy will be further discussed.

## CAF-tumor cell interaction regulates tumor progression and therapeutic response

As a principal constituent of the tumor stroma, CAFs play a central role in cross-communication between cells in tumors. In this section, we will mainly focus on colorectal cancer representing solid tumors, in which the transcriptional signatures of CAFs rather than tumor cells were robustly associated with poor disease prognosis and relapse across the various classifications [5, 6]. In the consensus molecular subtype (CMS) classification, which described a thoroughly studied and robust stratification strategy for CRC in large patient cohorts, CAFs were closely associated with CRC development [7, 8]. Subtype 4 (CMS4), the mesenchymal subtype, with overrepresentation of the stromal signature, predicted more aggressive tumor stages and worse prognosis.

### Secreted effectors and oncogenic signaling

The protumorigenic function of CAFs in CRC can be mainly exerted via secreted effectors such as growth factors, cytokines, chemokines, or exosomes, including the transforming growth factor β (TGF-β) family, interleukin (IL)-6, IL-8, IL-11, Wnt, hepatocyte growth factor (HGF), IL-17A, Netrin-1, leukemia inhibitory factor (LIF), secreted glycoprotein stanniocalcin-1 (STC1), fibroblast growth factor 1 (FGF1), stromal cell-derived factor-1 (SDF-1), and bone morphogenetic proteins (BMPs) [9–15].

**Fig. 1:**
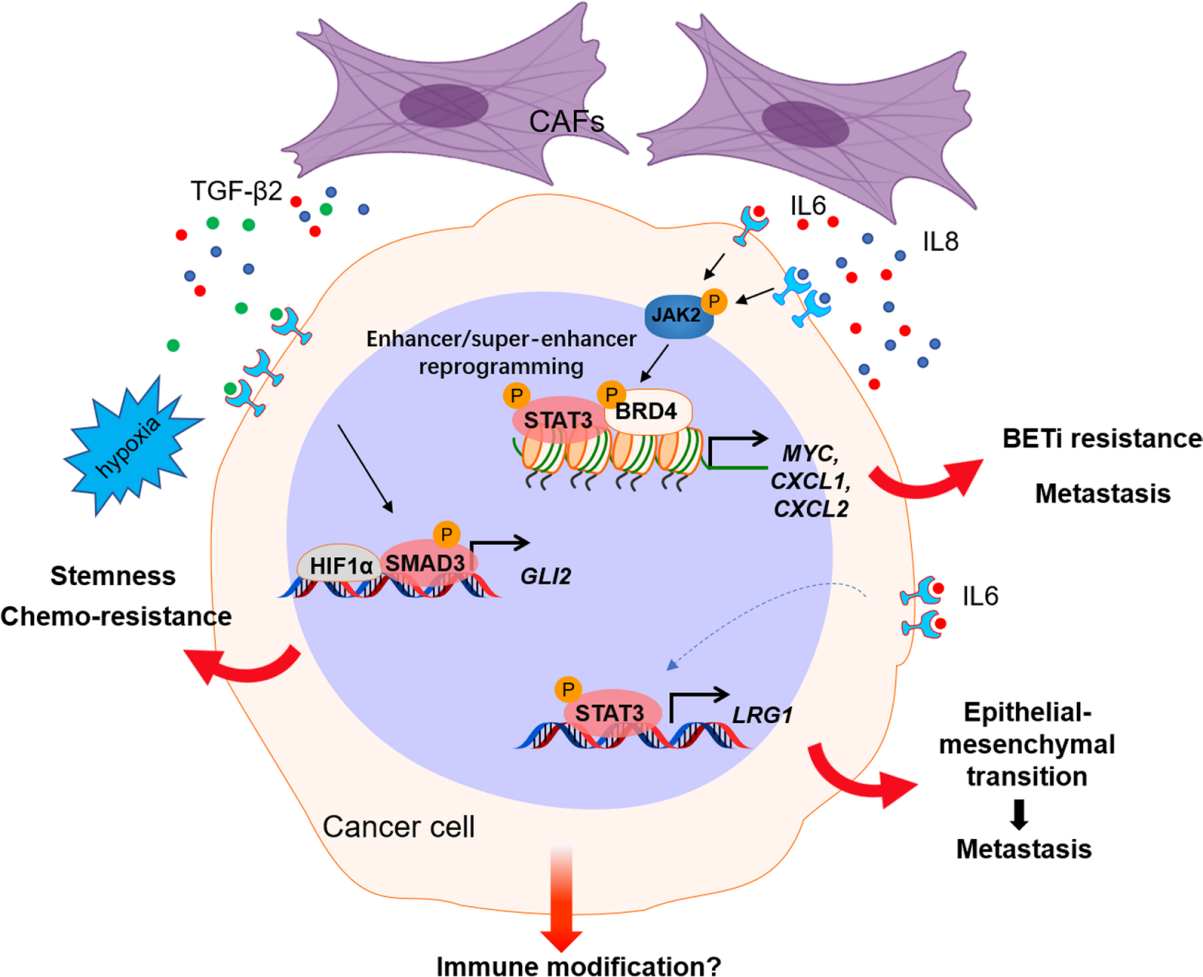
CAFs regulate tumor progression and therapeutic response. Our recent work demonstrated that CAFs promote tumor progression and therapeutic resistance through diverse mechanisms. CAFs secrete IL6 and IL8, which activate the JAK2-STAT3 pathway. JAK2-dependent phosphorylated BRD4 interacts with STAT3 to modulate chromatin remodeling (enhancer/super-enhancer reprogramming) and promote oncogene expression, leading to BETi resistance and tumor progression. IL6 also induces LRG1 expression through STAT3-dependent transactivation, which promotes EMT and metastasis. CAF-secreted TGF-β2 induced the expression of GLI2, which synergizes with hypoxia-induced HIF1α to promote CRC stemness and chemoresistance

In a noncontact coculture system, conditioned medium from CAFs rather than normal fibroblasts was found to promote the proliferation [16], migration, and invasion [17, 18] of CRC cells. TGF-β is one of the most important cytokines secreted mainly by CAFs and is highly expressed at tumor invasive margins. Interestingly, prominent TGF-β activation was also found in CRC subtype CMS4 [7]. The activation of TGF-β/Smad2 signaling in CAFs stimulated by CRC cells enhances the expression of α-SMA and differentiation of CAFs into a myofibroblastic phenotype, resulting in the expression of invasion-related proteins such as matrix metalloproteinases (MMPs) [19]. By developing patient-derived models to dissect the microenvironmental interaction between CAFs and tumor cells, we also described CAF-secreted TGF-β2, a member of the TGF-β family, inducing the expression of GLI Family Zinc Finger 2 (GLI2), an important effector of Hedgehog signaling, as a predominant pathway to promote CRC stemness and chemoresistance [20] (Fig. 1). Endoglin, a transmembrane accessory receptor of TGF-β that is expressed in CAFs in CRC and its metastatic specimens, is implicated in CAF-mediated invasion and metastasis via TGF-β signaling activation [21]. Additionally, integrin β6 expressed by CRCs was able to increase CAF activation through active TGF-β, and activated CAFs were found to promote CRC cell invasion [11].

Interleukin-6/signal transducer and activator of transcription 3 (IL-6/STAT3) signaling is a crucial and well-known pathway mediating malignant progression in CRC [22, 23]. CAFs in the tumor microenvironment play an active role in maintaining STAT3 activation in CRC. Heichler et al. found that the level of p-STAT3 activated by CAF-secreted IL-6/IL-11 was closely correlated with CRC patient survival [24]. TGF-β-stimulated CAFs activate STAT3 signaling in cancer cells, mediating tumor metastasis through the secretion of IL-11 [25]. Furthermore, STAT3 activation in fibroblasts promotes the expression of periostin, a multifunctional extracellular matrix protein, which ultimately facilitates CRC development by Integrin-FAK-Src pathway-mediated YAP/TAZ activation [26]. More recently, our work demonstrated that IL-6 could promote epithelial-mesenchymal transition (EMT), migration, and invasion of CRC cells through upregulation of leucine-rich α-2 glycoprotein 1 (LRG-1), which was found to be a direct transcriptional target of STAT3 [18]. Blocking the IL-6/LRG-1 axis remarkably attenuates metastasis in the xenograft CRC mouse model. IL-6-activated STAT3 in CAFs also regulates transcriptional patterns associated with angiogenesis. In a genetically engineered mouse model, the constitutive activation of STAT3 in fibroblasts promotes CRC growth, which is blocked by the inhibition of proangiogenic signaling [24]. It was also reported that the secretion of IL-6 from CAFs promotes angiogenesis by enhancing the production of the key angiogenic factor, vascular endothelial growth factor A (VEGFA) from endothelial cells [27]. IL-6 was also reported to promote colorectal cancer stem-like properties via induction of fos-related antigen 1 (FRA1) deacetylation [28]. Thus, targeting the microenvironment through STAT3 and its related signaling may provide a promising therapeutic opportunity for CRC treatment. Sanchez-Lopez et al. reported that targeting insulin-like growth factor-1 receptor (IGF-1R) and STAT3 decreased CRC proliferation and increased apoptosis by inhibiting CAF activation and inflammation [29].

The Wnt/beta-catenin signaling, one of the most activated pathways in CRC, was observed preferentially in tumor cells located close to stromal myofibroblasts. Myofibroblast-secreted factors, specifically hepatocyte growth factor (HGF), activate beta-catenin-dependent transcription and subsequently restore the cancer stem cell phenotype in more differentiated tumor cells both in vitro and in vivo [30]. In addition, Straussman et al. found that CAF-secreted HGF activates the MAPK and PI3K-AKT signaling pathways, resulting in resistance to RAF inhibitors in BRAF-mutant cancer cells [31]. In addition, CAF-derived Wnt2 can increase tumor angiogenesis [32] through the upregulation of some proangiogenic proteins and promote cell invasion and migration in CRC [33].

### Epigenetic regulation

Accompanying extensive regulation of signaling transduction and the tumor cell transcriptome, rewiring of the epigenetic landscape in tumor cells or CAFs can also modulate the extent and quality of the antitumor treatment response and overall disease outcome. Agrawal et al. discovered that fibroblasts promote cell proliferation and variably affect the expression of DNA methylation-regulating enzymes, hence improving decitabine-induced demethylation in CRC cells and increasing their stemness [34]. Bromodomain-containing protein 4 (BRD4), an epigenetic reader of histone acetylation, is highly expressed in different types of tumor cells, and it can protect these tumor cells against targeted therapy [35–38] and immunogenic cell death [39–41]. Our recent work demonstrated a tumor microenvironment mechanism by which CAF-associated inflammatory paracrine IL6/IL8-JAK2 signaling induces BRD4 activation by phosphorylation in CRC, leading to chromatin reprogramming through increased enhancer and super-enhancer activity. Interestingly, the chromatin remodeling induced by CAF-derived IL6/IL8 was established through the convergence of p-BRD4 and STAT3 co-occupancy on a set of crucial oncogenes associated with tumor growth and metastasis, such as MYC, C-X-C motif chemokine ligand (CXCL)1, and CXCL2. Additionally, noncoding RNAs are also involved in CAF-mediated tumor progression and therapeutic resistance. Several studies have reported that CAFs exert their roles by directly transferring exosomes to CRC cells, leading to a significant increase in noncoding RNA levels in CRC cells [42–44]. It was found that all CAF-derived lncRNA CCAL (colorectal cancer-associated lncRNA) [44], lncRNA H19 [43], and miR-92a-3p [42] transferred via exosomes can activate the Wnt/β-catenin pathway in CRC, then suppress cell apoptosis, promote cell stemness, and/or confer resistance to chemotherapy.

In addition to cancer cells, CAFs are also extensively regulated by epigenetic mechanisms in the TME. Leukemia inhibitory factor (LIF) is a cytokine highly expressed in CRC cells that can inhibit cell apoptosis and promote chemoresistance via the activation of STAT3 signaling [45]. Interestingly, LIF was also reported to activate quiescent CAFs by an epigenetic switch. Mechanistically, DNA methyltransferase 3 beta (DNMT3B), a de novo DNA methyltransferase that is activated in a LIF/STAT3-dependent manner, can methylate CpG sites and silence the expression of SHP-1 phosphatase, leading to the activation of CAFs [46]. Adenosine-to-inosine (A-to-I) RNA editing is a newly described epigenetic modification that is thought to represent a crucial carcinogenic mechanism in human malignancies. A study with a large cohort of 627 colorectal cancer (CRC) specimens by Takeda et al. revealed that adenosine deaminase acting on RNA (ADAR), the key enzyme involved in A-to-I RNA editing, was upregulated in both cancer cells and cancer-associated fibroblasts, which increased the RNA edition level of antizyme inhibitor 1 (AZIN1). Interestingly, conditioned medium from cancer cells led to both induction of ADAR1 expression and activation of AZIN1 RNA editing in CAFs, resulting in the increased invasive potential of CAFs within the TME in the colon [47].

These studies clearly showed that the tumor microenvironment is a comprehensive ecosystem in which intimate communication between cancer cells and CAFs fundamentally regulates tumor development and progression. These studies also strengthened that the therapeutic strategies mainly targeting tumor cells might be insufficient to achieve a curative outcome in cancer, which has been repeatedly observed in clinical practice. The tumor stroma supports cancer cell survival and drug resistance after exposure to these tumor-targeting therapies, leading to fatal progression. Interestingly, Lotti et al. discovered a considerable increase in the number of CAFs in CRC patients when chemotherapy was given. These CAFs maintain cancer-initiating cell self-renewal and lead to increased resistance to chemotherapy [48]. Thus, targeting CAFs must be considered to eliminate cancer. Notably, multiple strategies have been developed in preclinical and clinical models to target CAFs and their related pathways [49, 50]. Nevertheless, targeting CAFs alone is unlikely to be efficient in eliminating the whole tumor. Combination strategies that co-target tumor cells and CAFs may elicit tumor eradication. This relies on both mechanistic studies dissecting the complex interplay among cells in the tumor and the discovery of reliable biomarkers to stratify patients who may benefit from the treatment.

It is worth noting that the abovementioned stromal mechanism to regulate tumor progression and therapeutic response also deeply modulates tumor immunity within the TME, which will be discussed below. For instance, in addition to our finding that CAF-secreted TGF-β signaling and hypoxic environment-induced HIF-1α synergistically induce GLI2 expression to regulate tumor stemness and chemoresistance, it is well known that TGF-β signaling plays a vital role in tumor immunity in the TME by repressing the antitumor functions of various immune cell populations, including T cells and natural killer cells [51]. Interestingly, GLI2 and HIF-1α have also been found to regulate T cell and NK cell infiltration and activity in tumors [52–59]. Again, intriguingly, in addition to directly regulating angiogenesis and metastasis [18, 60], LRG1 has been shown to promote the extravasation and activation of neutrophils as well as to regulate NETosis [61], which is implicated in tumor immune suppression and neutrophil extracellular traps (NETs)-dependent metastasis [62, 63]. Thus, a more comprehensive understanding of the communications within the tumor microenvironment is needed for cancer therapies.

## Stromal mechanisms reshaping the TME and tumor immune response

Immunotherapy, specifically immune checkpoint blockade (ICB), has been the most promising paradigm in cancer therapies in the past decade. According to the abundance of tumor-infiltrating lymphocytes, tumors were arbitrarily classified as "hot tumors" or "cold tumors" with high infiltrated or low infiltrated lymphocytes, respectively [64]. While ICB has shown effectiveness in multiple cancers, such as melanoma and lung cancer, most patients cannot benefit from the treatment, especially those with cold tumors, such as CRC and breast cancer. In these “cold tumors”, due to the lack or paucity of tumor T cell infiltration, ICB treatment rarely triggers a strong immune response, leading to ICB failure [65]. Based on the underlying mechanism of ICB action, several potential features are proposed to be related to immunotherapy response, including programmed death-ligand 1 (PD-L1) expression level, immune composition within the TME (immune score), neoantigens and tumor mutation burden [66]. These features of tumors are determined not only by the genetic status of tumor cells (such as genetic mutations related to tumor antigens and mutation burden) but also by the stromal mechanisms by which CAFs reshape the TME through interplay with immune cells. Meanwhile, the epigenetic mechanism in the TME controlling these events has also been extensively documented, implying that certain epigenetic alterations could be used as potential targets for immunotherapy.

### The interplay between CAFs and immune cells to modulate tumor immunity

**Fig. 2:**
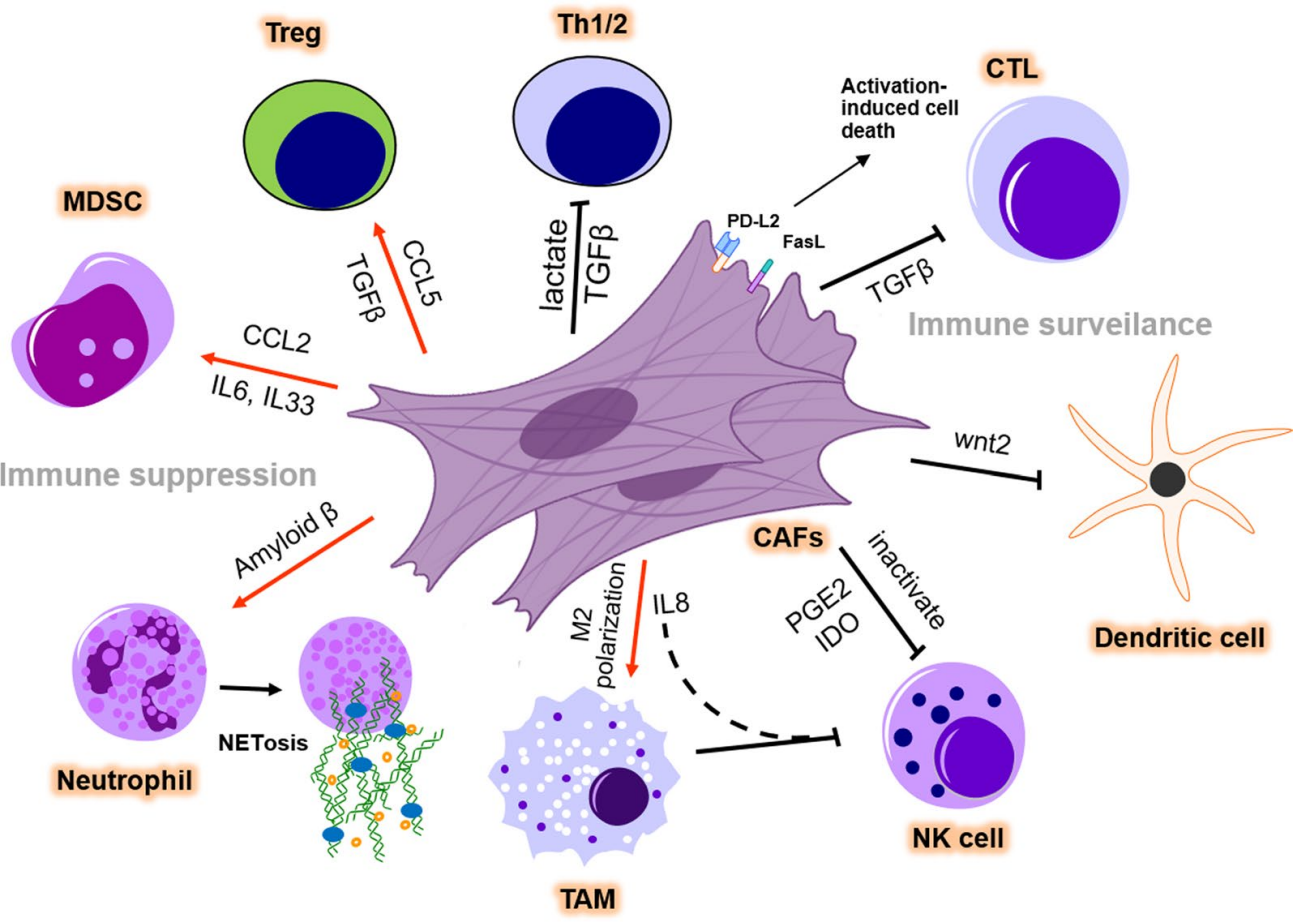
CAFs modulate the immunosuppressive microenvironment. CAFs promote immune suppression and abolish immune surveillance in the TME. CAFs secrete TGFβ and CCL5 to differentiate naïve T cells into Tregs and to recruit Tregs. CCL2, IL6, and IL33 secreted by CAFs help to recruit MDSCs and strengthen their immunosuppressive function. CAFs promote NETosis and M2 polarization of TMAs in the TME by releasing amyloid β or IL8. However, TGF-β secreted by CAFs suppresses Th cell function and reduces CTL infiltration. The expression of PD-L2 and FasL induces AICD in CTLs. CAFs can suppress the DC-mediated antitumor T cell response and inactivate NK cell-mediated tumor killing by PGE2 and IDO secretion. TME: tumor microenvironment; Th: T helper cell; Treg: regulatory T cell; MDSC: myeloid-derived suppressor cell; TAM: tumor-associated macrophage; NK cell: natural killer cell; AICD: activation-induced cell death

Recent studies have suggested that CAFs in the TME are linked to immunotherapy response by diverse mechanisms. For instance, CAFs and secreted ECM serve as a physical barrier to prevent drug delivery and infiltration of immune cells, thus restraining the effectiveness of immunotherapy [67, 68]. Moreover, the induction of immune checkpoint molecules such as PD-L1, PD-L2, and B7-H3 by CAF-secreted factors, exosomes in cancer cells or CAFs themselves substantially induce T cell exhaustion and deactivation, leading to intrinsic resistance to immunotherapy [69]. Additionally, cytokines such as IL-1β, IL-6, and TGF-β that can be produced by activated immune cells have been broadly implicated in CAF activation [19, 70, 71]. By interacting with immune cells such as T lymphocytes, myeloid-derived suppressor cells, dendritic cells, and others within the TME, CAFs can establish the so-called immunosuppressive microenvironment (Fig. 2).

#### CAFs and T lymphocytes

T lymphocytes function as essential modulators mediating the immune response, which comprises distinct subtypes such as cytotoxic CD8+ T lymphocytes (CTLs), Fox3p + regulatory T cells (Tregs), and CD4+ T helper (Th) cells. CTLs, the most critical immune cells of antitumor immunity, are substantially modulated by CAFs to reduce their infiltration, growth, and antitumor activity. CAF-secreted TGF-β inhibits the expression of cytolytic genes in CTLs, which are responsible for CTL-mediated tumor cytotoxicity [72]. Surprisingly, Lakins et al. found that CAFs isolated from murine melanoma and lung tumors can directly participate in antigen presentation, leading to antigen-mediated activation-induced cell death (AICD) of tumor-reactive CD8+ T lymphocytes via engagement of PD-L2 and Fas ligand to promote cancer immune evasion [73]. Furthermore, CAFs were reported to markedly stimulate Treg cell migration and increase their infiltration into tumor sites in CRC [74]. CAF-derived secreted factors such as TGF-β or CCL5 are also responsible for the recruitment of Tregs and differentiation of naïve T cells to Tregs, ultimately inducing immune suppression [75–77].

Several studies have indicated the significant influence of CAFs on Th cell polarization. For example, lactate release from CAFs reduced the percentage of antitumoral Th1 cells and concomitantly increased Tregs, thus driving immunosuppression in prostate cancer [78]. As one of the most frequently secreted cytokines by CAFs, TGF-β can suppress type 2 immunity by repressing Th2 cell responses in cancer [79].

#### CAFs and MDSCs

Myeloid-derived suppressor cells (MDSCs) are well documented for their immunosuppressive role in the TME. C–C motif chemokine ligand 2 (CCL2) released from CAFs in liver tumors was reported to promote the recruitment of MDSCs through the activation of STAT3 [80]. Similarly, CAF-produced IL-6 and IL-33 were able to educate MDSCs in the TME via hyperactivation of 5-lipoxygenase (5-LO), thus potentiating the capability of MDSCs to enhance cancer stemness [81]. Whereas, Yang et al. found that nonalcoholic fatty liver disease (NAFLD)-associated hepatocellular carcinoma (HCC) expressed low levels of CCL2 as well as other cytokines, such as CCL4, CXCL2, and CXCL6, compared with nontumor tissues [82]. Although somehow contradictory to the immunosuppressive function of CCL2, this study demonstrated that CCL4, a crucial chemokine for T cell migration, is more responsible under this circumstance. Interestingly, pharmacological inhibition of histone deacetylase 8 (HDAC8), a histone H3 lysine 27 (H3K27)-specific isozyme overexpressed in a variety of human cancers, increased global and enhancer acetylation of H3K27 to reactivate the production of CCL4 by HCC cells, thus dampening HCC tumorigenicity in a T cell-dependent manner.

#### CAFs and other immune cells

Many reports have also uncovered the importance of CAFs in mediating tumor immune evasion by regulating innate immune cells, such as dendritic cells (DCs), tumor-associated macrophages (TAMs), neutrophils, natural killer (NK) cells, and myeloid cells. In CRC, CAF-secreted Wnt2 led to evasion of immune surveillance by suppressing the DC-mediated antitumor T cell response through the SOCS3/p-JAK2/p-STAT3 signaling cascades [83]. Moreover, a comprehensive map to elaborate the interaction between diverse types of cells in the TME of CRC has been depicted recently by taking advantage of scRNA-seq using clinical samples [84]. Of note, SPP1+ TAMs displayed direct interaction with CAFs, which might be due to the binding of MMP2 produced from CAFs and SDC2 that was preferentially expressed in SPP1+ TAMs [84]. In line with this, another work by Zhang et al. also confirmed that CAFs promoted TAM infiltration and subsequent M2 polarization in CRC via IL-8 [85]. Furthermore, TAMs could synergize with CAFs to suppress NK cell killing ability, therefore promoting CRC progression. Neutrophils release histone-bound nuclear DNA and cytotoxic granules as extracellular traps (NETs). A novel finding demonstrated that CAF-secreted amyloid β drives the formation of tumor-associated NETs (t-NETs), thus supporting tumor progression [86]. More interestingly, it was also observed that t-NETs could reciprocally activate CAFs by promoting their expansion, contractility, and deposition of matrix components [86]. CAFs inhibit NK cells through a variety of mechanisms. CAFs, for example, reduce the expression of NK cell-activating receptors, including NKp30 and NKp44, and transition NK cells into an inactivated state by secreting prostaglandin E2 (PGE2) and indoleamine 2,3-dioxygenase (IDO) [87, 88]. Surprisingly, NK cells can enhance this suppressive loop by boosting the release of PGE2 by CAFs [87]. It has also been reported that CAFs indirectly decrease NKG2D-dependent cytotoxic activity and interferon (IFN) secretion of NK cells by reducing the ligands of NK-activating receptors on melanoma cells [89]. Previous research has shown that various myeloid cell subsets expand in CRC cancers. However, these tumor-infiltrating myeloid cells have both pro- and anti-tumor roles in CRC progression. Salman et al. discovered that CD33+ myeloid cells from patients with advanced stages expressed more pro-angiogenic and hypoxia-related genes but fewer immune and inflammatory response genes compared to those with early-stage diseases [90]. This study implies that immune cell recruitment and activation could be compromised under the TME, which dynamically evolves along with tumor progression.

These works highlighted that CAFs and immune cells formed an intimate connection within the TME, implying a promising potential strategy to reshape the immune microenvironment by perturbing crosstalk between the two cell populations.

### Epigenetic mechanisms in the TME modulate immunotherapy efficacy

**Fig. 3:**
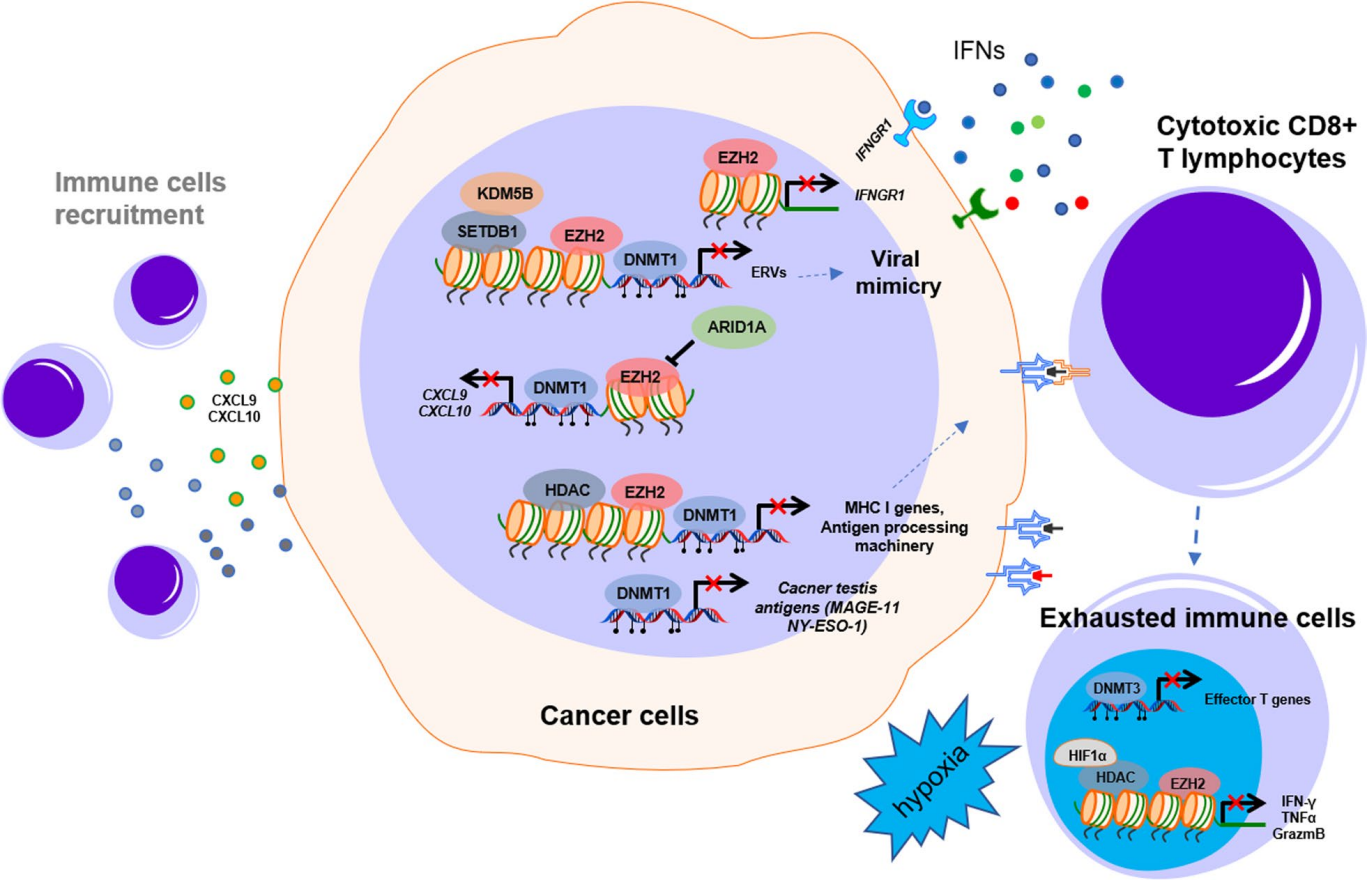
Epigenetic regulation of the immune response in the tumor microenvironment. DNA methylation and histone modification regulate the tumor immune response in the TME. The epigenetic mechanisms of DNA methylation induced by DNMT, transcriptional suppression by EZH2, and HDAC play crucial roles in immune-related signal inactivation, immune cell recruitment, antigen processing and presentation, and immune cell exhaustion by suppressing the expression of ERVs, MHC I genes, antigen processing machinery, and cancer testis antigens in the TME. TME: tumor microenvironment; IFNs: interferons

The complex interplay between stroma, immune, and cancer cells alters the epigenome of each other, which is important for antitumor immunity. The idea of converting noninflamed cold tumors into hot tumors using epigenetic intervention may help to achieve a better response to immunotherapy [91]. Early testing of the therapeutic potential of combining epigenetic agents and immunotherapies showed elevated immune-related gene expression and a durable response to anti-CTLA4 or anti-PD1 treatment [92–94]. Epigenetic modifications of immune-related genes may strengthen immune surveillance and increase the efficacy of immunotherapy by three key mechanisms (Fig. 3): (1) activating immune pathways or reprogramming the tumor microenvironment to counteract immunosuppression. (2) Increasing the tumor antigenicity by enhancing the processing and presentation of tumor antigens (3) reversing the exhaustion of infiltrated cytotoxic immune cells in the tumor.

#### Modulation of key immune signaling pathways in the TME

As evidenced by the existence of an IFN-responsive gene profile in some tumors, an inflamed "hot" TME is compatible with effective IFN-mediated antitumor immune responses. IFN signaling, including type I IFN (IFNα and IFNβ) and type II IFN (IFN-γ), is a well-controlled molecular network that plays pivotal roles in tumor immunity.

Type I interferons control the development of innate and adaptive immune responses by activating intracellular viral defense pathways. Viral double-stranded DNA (dsDNA) or dsRNA can activate the production of type I interferons when captured by their sensors. It is worth noting that the cytosolic dsDNA sensing pathway, especially the cyclic GMP-AMP synthase and stimulator of interferon genes (cGAS-STING) pathway, is usually epigenetically silenced in human cancers through DNA hypermethylation at their promoter regions [95–98]. The reactivation of ancient endogenous retroviruses (ERVs) and retrotransposons in our genome that are typically silenced (so-called viral mimicry) has been emerging as a robust strategy to boost the immune response in cancer [99, 100] by inducing type I IFN activation after being recognized by sensors of dsRNA, such as RIG-I and MDA5. Recent studies have shown that ERVs can be reinvigorated by drugs targeting epigenetic modulators, including DNMTs, HDACs, or HMTs. In many cancers, including CRC, DNA methyltransferase inhibitors (DNMTis) can induce dsRNA expression mainly derived from ERVs and subsequently trigger cytosolic sensing of dsRNA, causing a type I interferon response and apoptosis [93, 101]. Interestingly, similar to DNMT1 inhibition, ablating the histone demethylase LSD1, which is elevated in diverse cancers, improves tumor immunogenicity by simultaneously activating the dsRNA-IFN pathway by stimulating ERV expression and downregulating the RNA-induced silencing complex (RISC) [102]. These findings may provide an opportunity to reactivate the pathway and promote the immune response by targeting specific epigenetic regulators.

Moreover, Morel et al. demonstrated that EZH2 represses the production of dsRNA and genes implicated in the IFN response, antigen presentation, and T-cell attraction through its catalytic function in prostate cancer [103]. As a histone methyltransferase, SETDB1 was first found to keep silencing the transposable elements (TEs) that lead to the production of dsRNAs in acute myeloid leukemia (AML) [104]. SETDB1 is located at a frequently amplified chromosome area in many other solid tumors, chromosome 1q21.3, which was also implicated in worse tumor prognosis in breast cancer [105]. The amplification of SETDB1 (1q21.3) in tumors is associated with immune exclusion and resistance to immune checkpoint blockade [106]. SETDB1 loss derepresses latent TE-derived regulatory elements, immunostimulatory genes, and TE-encoded retroviral antigens in these regions and triggers TE-specific cytotoxic T cell responses in vivo. Using melanoma and colon cancer as models, Zhang et al. uncovered that KDM5B—an H3K4 demethylase—recruits the H3K9 methyltransferase SETDB1 to repress endogenous retroelements in a demethylase-independent manner [107]. Although it remains to be further determined whether these epigenetic regulations commonly occur in colon cancer, the viral mimicry induced by epigenetic intervention provides an apparent strategy to trigger a robust IFN response and antitumor immunity within the TME. Strikingly, ERV regulation also determines T helper cell lineage integrity. In immune T helper cells, SETDB1 controls the deposition of the restrictive H3K9me3 mark at a restricted and cell-type-specific set of endogenous retroviruses positioned in the vicinity of genes implicated in immunological processes [108]. These retrotransposons operate as Th1 gene enhancers or influence Th1 gene cis-regulatory elements. By suppressing a range of ERVs to shape and govern the Th1 gene network, H3K9me3 deposition by SETDB1 ensures Th cell lineage fidelity.

IFN-γ binds to interferon gamma receptors (IFNGRs) and activates the Janus kinase (JAK)-signal transducer and activator of transcription (STAT) signaling pathway, which modulates the immune response by activating an IFN-stimulated gene (ISG) transcriptional program. The presence of an IFN-γ-responsive gene signature predicts a better response to immunotherapy compared with tumors lacking the IFN-γ signature [109].

Epigenetic histone modifications and DNA methylation are closely involved in the regulation of the IFN-γ signaling pathway in colorectal cancer. Tumor production of CXCL9 and CXCL10, which are Th1-type chemokines, can be repressed by either enhancer of zeste homolog 2 (EZH2, a core of the PRC2 complex)-mediated histone H3 lysine 27 trimethylation or DNA methyltransferase 1 (DNMT1)-induced DNA methylation, subsequently resulting in less recruitment of IFN-γ-producing immune cells [110]. Conversely, ARID1A, a core member of the SWItch/Sucrose Non-Fermentable (SWI/SNF) complex, promotes tumor expression of CXCL9 and CXCL10 [111]. Genetic deficiency in ARID1A has been reported to result in a reduction in chromatin accessibility at these chemokine loci in colorectal cancer cells. ARID1A interacts with EZH2 through its carboxyl terminus, preventing EZH2 from inhibiting IFN signaling-mediated gene expression. Moreover, our previous work discovered that EZH2 can suppress the IFN-γ signaling pathway by directly silencing the expression of interferon-γ receptor 1 (IFNGR1) [112] and ISG activation [113], which led to cancer cells being insensitive to IFN-γ treatment or resistant to trastuzumab treatment, respectively.

#### Improvements to tumor antigenicity

Aberrant epigenetic mechanisms driving the dysregulation of genes involved in the processing or presentation of tumor antigens, essential for T cell activation, are a recurring characteristic of cancer cells escaping from immune surveillance. In addition to activating IFN signaling, DNMTis such as 5-azacytidine, decitabine, and guadecitabine, which induce global hypomethylation, significantly boost the expression of MHC class I genes and PD-L1 [114, 115]. Additionally, DNMTi can also increase the expression of cancer-testis antigens (CTAs), promising immunotherapy targets such as MAGE-11 and NY-ESO-1 that are expressed in early embryonic cells but suppressed in mature somatic cells due to promoter CpG island DNA methylation [116, 117]. In cancer cells, deacetylation of histone lysine residues is frequently linked to hypermethylated and repressed genes. Histone deacetylase inhibitors (HDACis), such as trichostatin A (TSA), restore gene expression by targeting these regions. HDACis have been shown to boost the expression of various antigen processing machinery components, such as TAP-1, TAP-2, LMP-2, and tapasin. Treatment of metastatic cancer cells with TSA increases MHC class I expression on the cell surface, which functionally translates to increased vulnerability to killing by antigen-specific CTLs [118]. PRC2 was also reported to silence the MHC-I antigen processing and presentation pathway and evade immune surveillance. Pharmacological inhibition of EED or EZH2 and EZH1 reverses the silencing of these pathways, leading to the reestablishment of effective T cell-mediated antitumor immunity.

#### Reversed immune exhaustion

Tumor-infiltrating lymphocytes, particularly cytotoxic CD8+ T cells (CTLs), often display dysfunction and exhaustion due to the persistent existence of antigen stimulation and other factors in the TME, such as hypoxia and metabolic stress [119]. They frequently lose the capacity to produce cytokines such as tumor necrosis factor-α, IFN-γ, and interleukin (IL)-2 but retain the expression of inhibitory receptors such as programmed cell death protein (PD)-1, lymphocyte-activation gene (LAG)-3, or T cell immunoglobulin and mucin-domain containing (TIM)-3 [120, 121]. Specific chromatin-accessible areas linked with an altered transcriptional profile are found in CD8+ T cell exhaustion, including enrichment for genes in interferon signaling, PD-1 signaling and the cytokine IL-10 response [122]. Immune checkpoint blockade, such as anti-PD-1 antibody treatment, has been shown to partially reverse CD8+ T cell exhaustion; however, extensive epigenetic reprogramming during T cell exhaustion, which differs substantially from that of effector and memory T cells, limits the durable success of immunotherapies [123]. By characterizing the critical epigenetic reprogramming mechanisms of T cell exhaustion, the exhaustion status may be reversible [124–127]. Ghoneim et al. demonstrated that epigenetic changes introduced by the DNA methyltransferase DNMT3A are needed to acquire an exhausted phenotype[126]. DNMT3A methylates thousands of genes de novo, many of which are critical for effector CD8+ T cell function. A study of exhausted CD8+ T cells in humans and a chronic viral infection mouse model by Sen et al. revealed that a state-specific epigenetic landscape organized into functional modules of enhancers is required for exhaustion [124]. Using an in vitro system that models human T cell exhaustion, our data recently reported that hypoxia in the TME induces transcriptional suppression of the immune effectors IFN-γ, tumor necrosis factor α (TNFα), and granzyme B, resulting in immune effector cell dysfunction and resistance to immunotherapy [128]. Furthermore, the chromatin remodeling enforced by HIF1α interaction with HDAC1 and subsequent dependence on PRC is identified as a crucial epigenetic mechanism conferring immune effector suppression. In addition, under continuous stimulation with tumor antigen, hypoxia further induces TIM-3 and ITGIT to potentiate T cell exhaustion in a HIF-1α-independent manner. In addition, microenvironmental stressors coordinated with T cell receptor stimulation, and PD-1 signaling can promote terminal exhaustion of T cells through epigenetic reprogramming as a result of mitochondrial dysfunction [129].

## Implications of epigenetic modulators in cancer intervention

Many studies have focused on evaluating combinations of immunotherapies with various therapies, including chemotherapy, radiation therapy, and targeted therapy, to increase the infiltration of CTLs [130]. With the idea of converting "cold tumors" to "hot tumors", epigenetic therapy offers a unique opportunity to remodel the TME from immunosuppressive to immunopermissive by regulating stromal and immune cells via multiple mechanisms [91]. Multiple preclinical studies have discovered that epigenetic agents can reinvigorate the immune response in various tumor types. As discussed in the previous sections, DNA hypomethylating agents such as DNMTi (5-AZA), EZH2 inhibitors, or HDACi (TSA) can improve the efficacy of ICB by reducing immune suppression through the initiation of the type I IFN response via dsRNA production. 5-AZA increased the infiltration of both CD8+ T and natural killer (NK) cells and reduced the percentages of macrophages and MDSCs in the TME. Interestingly, Zhou et al. recently revealed that p53 activation by MDM2 inhibitors induced the type I IFN response, abolishing tumor immune evasion and promoting antitumor immunity in an LSD1- and DNMT1-dependent manner [131]. The importance of p53 during cancer progression is unequivocal since more than half of all sporadic cancers show p53 dysfunction. Furthermore, the MDM2 inhibitor ALRN-6924 induced a viral mimicry response and tumor inflammation signature genes in melanoma patients, which provided a rationale for the synergistic strategy of MDM2 inhibitors and immunotherapy. Additionally, in mouse mammary tumor models (MMTV-rtTA/tetO-HER2, MMTV-PyMT) and patients with breast and colon carcinoma, treatment with CDK4/6 inhibitors reduced DNMT1 expression, resulting in hypomethylation of immune-related genes, enhancing antitumor immunity by both promoting antigen presentation and reducing Treg cell expansion [132]. These events ultimately promoted clearance of tumor cells by cytotoxic T cells, which could be further improved by the addition of immune checkpoint blockade (anti-PD-L1), thus opening a new avenue to treat cancer by combination regimens comprising CDK4/6 inhibitors and immunotherapies.

Interestingly, many epigenetic modulation agents play roles in different aspects of immune modulations. For example, DNMTi can initiate the type I IFN response and has functions in regulating tumor antigen presentation. HDACis could restore tumor antigen expression and reverse T cell exhaustion. Although these functions may be played under different contexts, it is interesting to determine how to leverage them to augment antitumor immunity. In some circumstances, the combination of different epigenetic agents plus ICB may confer the best antitumor effect. For example, a triple combination of DNMTi/HDACi plus the immune checkpoint inhibitor α-PD-1 provides the most prolonged overall survival in an ovarian cancer model [133]. Similarly, histone deacetylase 6 (HDAC6) inhibitors with enhanced antitumor immunity of anti-PL-L1 immunotherapy were recently developed for melanoma treatment [134]. A concern is that many epigenetic inhibitors have been shown to limit T cell growth, which could compromise the long-term effectiveness of immunotherapy that relies on a persistent T cell population. Inhibition of EZH2, for example, has been shown to impair T cell function [135]. EZH2 is required to generate and maintain memory T cells, which are responsible for effector T cell production and antitumor activity. In conclusion, more research is needed to determine whether the benefit of combining epigenetic therapy and immunotherapy is dependent on the type of cancer or other circumstances. Recently, many strategies combining epigenetic therapy and immunotherapy are being evaluated in numerous clinical trials (summarized in Table 1), which may improve clinical practice in the future.

**Table 1:** Clinical trial combining epigenetic targetings with immunotherapies

Type of epigenetic drugs	Epigenetic drug (targets)	Immunotherapy	Cancer type	Trial ID
DNMT inhibitors	Azacytidine	Nivolumab (anti-PD1)	AML, NSCLC, Osteosarcoma	NCT02397720 [136]; NCT03825367 [137]; NCT01928576 [138]; NCT03628209 [139]
		Pembrolizumab (anti-PD1)	AML,CRC/microsatellite-stable CRC,HNSCC, Melanoma,MDS,NSCLC, PDAC,Ovarian primary peritoneal or fallopian tube cancer	NCT02546986 [140]; NCT02959437 [141]; NCT02845297 [142]; NCT03769532 [143]; NCT02260440 [144]; NCT02512172 [145]; NCT03094637 [146]; NCT02816021 [147]; NCT03264404 [148]; NCT02900560 [149]
		Visilizumab(anti-PD1)	Relapsed Adult AML	NCT04722952 [150]
		Atezolizumab (anti-PDL1)	MDS	NCT02508870 [151]
		Avelumab (anti-PDL1)	AML	NCT02953561 [152]; NCT03390296 [153]
		Durvalumab (anti-PDL1)	MDS, AML, NSCLC, Head and neck cancer, Breast cancer	NCT02775903 [154]; NCT02117219 [155]; NCT02250326 [156]; NCT03019003 [157]; NCT02811497 [158]
		Ipilimumab (anti-CTLA4)	MDS	NCT02530463 [159]
		Tremelimumab (anti-CTLA4)	MDS, Head and neck cancer	NCT02117219 [155]; NCT03019003 [157]
		Pembrolizumab and epacadostat (IDO-1 inhibitor)	Advanced Solid Tumors, Non-Small Cell Lung Cancer, Microsatellite-Stable CRC	NCT02959437 [141]
	Decitabine (DNMT1)	Camrelizumab (anti-PD1)	Hodgkin Lymphoma, PMBCL	NCT04514081 [160]; NCT03250962 [161]; NCT03346642 [162]
		Nivolumab(anti-PD1)	Non-small Cell Lung Cancer	NCT02664181 [163]
		Pembrolizumab (anti-PD1)	AML, MDS, CNS solid tumors, NSCLC, Breast cancer	NCT02996474 [164]; NCT03969446 [165]; NCT02957968 [166]; NCT03445858 [167]; NCT03233724 [168];
		Avelumab (anti-PDL1)	AML	NCT03395873 [169]
		pilimumab (anti-CTLA4)	MDS, AML	NCT02890329 [170]
	Decitabine(with Chidamide, a HDAC inhibitor)	Camrelizumab (anti-PD1)	Hodgkin Lymphoma	NCT04514081 [160]
	Guadecitabine(SGI-110)	Nivolumab (anti-PD1)	CRC	NCT03576963 [171]
		Pembrolizumab (anti-PD1)	NSCLC, CRPC	NCT02998567 [172]; NCT02901899 [173]
		Atezolizumab (Anti-PD-L1)	AML, MDS, urothelial carcinoma,Ovarian Carcinoma, Fallopian Tube Carcinoma, Primary Peritoneal Carcinoma	NCT02892318 [174]; NCT02935361 [175]; NCT03179943 [176]; NCT03206047 [177]
		Durvalumab (anti-PDL1)	Renal cancer, hepatocellular carcinoma, PDAC	NCT03308396 [178]; NCT03257761 [179]
		Ipilimumab (anti-CTLA4)	Metastatic Melanoma	NCT02608437 [180]
	Guadecitabine( with Mocetinostat HDAC inhibitor)	Pembrolizumab (anti-PD1)	NSCLC	NCT03220477 [181]
EZH2 inhibitors	Tazemetostat	Pembrolizumab (anti-PD1)	Urothelial carcinoma	NCT03854474 [182]
		Atezolizumab (anti-PDL1)	DLBCL	NCT02220842 [183]
	CPI-1205	Ipilimumab (anti-CTLA4)	Advanced solid tumours	NCT03525795 [184]
BET inhibitor	INCB057643	Pembrolizumab and epacadostat (IDO-1 inhibitor)	Advanced Solid Tumors, Non-Small Cell Lung Cancer, Microsatellite-Stable CRC	NCT02959437 [141]
	BMS-986158	Nivolumab (anti-PD1)	haematologic malignancies	NCT02419417 [185]
LSD inhibitor	INCB059872	Pembrolizumab and epacadostat (IDO-1 inhibitor)	Advanced Solid Tumors, Non-Small Cell Lung Cancer, Microsatellite-Stable CRC	NCT02959437 [141]
		Nivolumab (anti-PD1)	SCLC	NCT02712905 [186]
HDAC inhibitor	Chidamide with Decitabine	Immune checkpoint inhibitors(anti-PD1/PD-L1/CTLA4 antibodies)	Non-Hodgkin Lymphoma and advanced solid tumors	NCT05320640 [187]
	Entinostat	Nivolumab (anti-PD1)	PDAC	NCT03250273 [188]
		Pembrolizumab (anti-PD1)	Advanced solid tumours, Lymphomas, melanoma, bladder cancer, MDS	NCT03179930 [189]; NCT02936752 [190]; NCT03978624 [191]; NCT02437136 [192]; NCT02909452 [193]; NCT03765229 [194]
		Nivolumab and ipilimumab	Renal cell carcinoma	NCT02453620 [195]; NCT03552380 [196]
		Avelumab (anti-PDL1)	Ovarian cancer	NCT02915523 [197]
	Mocetinostat	Nivolumab (anti-PD1)	NSCLC	NCT02954991 [198]
		Pembrolizumab (anti-PD1)	NSCLC	NCT03220477 [181]
		Durvalumab (anti-PDL1)	NSCLC	NCT02805660 [199]
		Nivolumab and ipilimumab	Melanoma	NCT03565406 [200]
	Vorinostat	Pembrolizumab (anti-PD1)	HNSCC, NSCLC, renal or urothelial cell carcinoma, Breast cancer, Glioblastoma, DLBCL, Hodgkin Lymphoma	NCT02638090 [201]; NCT03426891 [202]; NCT03150329 [203]; NCT02538510 [204]; NCT02619253 [205]; NCT02395627 [206]

AML: acute myeloid leukaemia; BET: Bromodomain and extra-terminal; CNS: central nervous system; CRC: colorectal cancer; CRPC: castration-resistant prostate cancer; CTLA4: cytotoxic T lymphocyte-associated protein 4; DLBCL: diffuse large B cell lymphoma; DNMT: DNA methyltransferase; EZH2: enhancer of zeste homologue 2; HDAC: histone deacetylase; HNSCC: head and neck squamous cell carcinoma; MDS: myelodysplastic syndrome; NSCLC: non-small-cell lung cancer; PDAC: pancreatic ductal adenocarcinoma; PMBCL: primary mediastinal large B cell lymphoma; SCLC: small-cell lung cancer

## Concluding remarks

In summary, this review broadly discusses recent studies exploring the complex interaction networks across key cell components within the TME, which consist of CAFs, tumor cells, and immune cells. Reciprocal crosstalk between different cell populations ultimately determines tumor progression via diverse "intermediate massagers". Epigenetic dysfunction has emerged as a novel hallmark of cancer. Although in-depth research has indicated the critical influence of epigenetic regulation on cancer cells, rising evidence has pointed to the other appealing property of epigenetic modulators in reshaping the TME, especially from the perspective of creating a tumor-favor immunosuppressive condition. As comprehensively stated above, various epigenetic modulators contribute to immune evasion, and hence, targeting them with small molecules could boost the immune response. Thus, these findings present a promising strategy to combine epi-drugs with other therapies, such as immune checkpoint blockade (ICB) therapy, which requires an immune-permissive TME as the prerequisite for successful treatment. Moreover, while ICB therapy undoubtedly became one of the most powerful tools to treat multiple cancers with a durable response and acceptable toxicity, up to approximately 85% of patients displayed intrinsic or acquired resistance to ICB, which profoundly limits its utility in the clinic. Therefore, the identification of epigenetic markers that can predict patients benefiting from ICB treatment merits further investigation in the future.

## Data Availability

Not applicable.
